# Dynamic context-aware multi-modal deep learning for longitudinal prediction of Parkinson’s disease progression

**DOI:** 10.1038/s41598-025-31898-y

**Published:** 2025-12-11

**Authors:** Amin Dehghanghanatkaman

**Affiliations:** Department of Computer Science, Shahid Bahonar University, Kerman, 76169-13439 Iran

**Keywords:** Computational biology and bioinformatics, Engineering, Health care, Mathematics and computing, Neurology, Neuroscience

## Abstract

Accurately forecasting the progression of Parkinson’s disease (PD) motor symptoms in early-to-moderate stages is essential for timely intervention and personalized patient care but remains challenging due to heterogeneous and longitudinal symptom evolution. We present a novel dynamic context-aware multi-modal deep learning framework that predicts future motor symptom severity by integrating advanced voice biomarkers with signal processing techniques, clinical progression features, demographic metadata, and semantically enriched patient summary embeddings derived from comprehensive clinical narratives via state-of-the-art natural language processing. Leveraging bidirectional LSTMs augmented with multi-head self-attention, our architecture captures complex temporal dependencies while preventing information leakage. To ensure robust evaluation despite limited sample size (42 patients), we implemented repeated 5-fold cross-validation at the patient level (8 repetitions, 40 total folds), substantially exceeding standard evaluation rigor. Our approach achieves exceptional performance ($\hbox {R}^2$ = 0.9925 ± 0.0027, RMSE = 0.67 ± 0.19, MAE = 0.50 ± 0.15) with all 40 folds achieving $\hbox {R}^2$ > 0.989, significantly outperforming classical machine learning baselines ($p < 1 \times 10^{-5}$ and 0.002785) and all previously published methods on this dataset. Cross-validated ablation studies (240 total model trainings across 6 configurations) reveal that clinical features establish a strong baseline ($\hbox {R}^2$ = 0.9887 ± 0.0043), while text embeddings provide the largest incremental gain (3.82% RMSE reduction). Voice biomarkers contribute modestly to accuracy (2.72%) but substantially enhance stability (10-fold lower variability). The full multi-modal model achieves optimal performance (7.50% RMSE reduction vs. clinical-only) with the lowest variability (CV = 0.27%), demonstrating that dynamic cross-modal fusion enhances both accuracy and robustness. These findings, validated through 40 independent evaluations with each patient tested 8 times, demonstrate that integrating engineered temporal dynamics and contextual embeddings through advanced temporal modeling enables accurate longitudinal predictions of early-to-moderate PD progression. Complete code and implementation details are publicly available to ensure reproducibility.

## Introduction

Parkinson’s disease (PD) is a progressive neurodegenerative disorder characterized by a complex interplay of motor and non-motor symptoms that evolve over time, posing significant challenges for early diagnosis, individualized prognosis, and effective disease management^1^.

Longitudinal monitoring of PD progression is critical for optimizing therapeutic interventions and improving patient outcomes, yet accurately predicting disease trajectories remains a formidable task due to the heterogeneity of symptom evolution and the multifaceted nature of clinical data^1,2^.

Recent advances in digital health technologies have enabled the collection of rich, multi-modal datasets, including voice recordings, clinical assessments, and demographic information, that capture diverse aspects of PD pathology^3^. Voice analysis, in particular, has emerged as a promising non-invasive biomarker for PD, as vocal impairments often precede overt motor symptoms and reflect underlying neurodegenerative processes^4,5^. Numerous studies have demonstrated the utility of acoustic features such as jitter, shimmer, and harmonic-to-noise ratio for distinguishing PD patients from healthy controls and tracking disease severity^4,6^. However, most prior approaches rely on cross-sectional data or simple statistical models, limiting their ability to capture the temporal dynamics and individualized progression patterns inherent to PD^1,7^.

Deep learning methods, especially those leveraging recurrent neural networks (RNNs) and attention mechanisms, have shown considerable promise in modeling complex, longitudinal clinical data^8^. Multi-modal architectures that integrate heterogeneous data sources, such as structured clinical variables, time-series biosignals, and unstructured text, can enhance predictive performance by capturing complementary information and contextualizing patient trajectories^9,10^. Notably, recent works have applied transformer-based models and context-aware fusion strategies to improve the accuracy of disease progression prediction in neurodegenerative disorders^8,11^.

Despite these advances, several key challenges remain: (1) effectively integrating diverse data modalities while preserving temporal and contextual relationships; (2) mitigating data sparsity and missingness common in longitudinal clinical datasets; and (3) providing interpretable, patient-specific predictions that can inform clinical decision-making^12,13^. Addressing these gaps is essential for realizing the full potential of artificial intelligence in personalized PD management.

In this study, we propose a dynamic context-aware multi-modal deep learning framework for longitudinal prediction of Parkinson’s disease progression. Our approach integrates processed voice features,meta-data, temporal clinical markers, and semantically enriched textual patient summaries using a novel dynamic attention fusion mechanism. By leveraging advanced natural language processing (NLP) techniques to encode patient-specific clinical context and employing robust temporal modeling, our method aims to provide accurate, interpretable, and individualized forecasts of disease trajectories. We rigorously evaluate our model on the Parkinson’s Telemonitoring dataset^14^, a comprehensive longitudinal PD dataset and demonstrate its superiority over conventional approaches, highlighting the promise of multi-modal, context-aware deep learning in advancing precision neurology.

The remainder of this paper is organized as follows: Section (Methods) details our dynamic context-aware multi-modal deep learning framework, including data preprocessing, feature engineering, model architecture, and cross-validation strategy. Section (Results) presents experimental findings, including cross-validated performance metrics, baseline comparisons, and ablation studies. Section (Discussion) interprets model performance, addresses clinical implications, discusses limitations, and outlines future research directions. Section (Conclusion) summarizes key contributions. This structure follows standard scientific reporting conventions to facilitate reader comprehension.

## Literature review

PD is a complex, progressive neurodegenerative disorder characterized by heterogeneous symptom trajectories, making accurate longitudinal prediction of motor progression essential for personalized management. Recent advances in machine learning have increasingly focused on multimodal data fusion and temporal modeling to improve predictive accuracy and interpretability.

Longitudinal modeling of continuous Parkinson’s disease (PD) clinical scores, such as the MDS-UPDRS, is essential for understanding and forecasting disease progression. While much of the earlier literature focused on classification or early diagnosis, recent advances have shifted toward regression-based approaches that predict symptom trajectories over time. For instance, Lian et al. (2024) introduced a personalized progression modeling framework that integrates multimodal clinical and imaging data using a novel graph-based approach. Their model captures complex relationships across data types and enables accurate, individualized prediction of multiple PD symptom trajectories, highlighting the value of multimodal integration and graph learning for continuous outcome prediction^15^. Building on this, Huang et al. (2023) proposed a multi-task graph structure learning framework employing node clustering to enhance interpretability and prediction accuracy in PD modeling, primarily demonstrated on early diagnosis tasks but with potential applicability to longitudinal progression^16^. Complementing these machine learning innovations, large-scale longitudinal meta-analyses have established the value of regression modeling in quantifying progression rates and informing clinical trial design. Arshad et al. (2023), for example, analyzed historical PD trials to model longitudinal changes in UPDRS scores, providing critical benchmarks for future research and therapeutic development^17^.

In addition to graph-based and deep learning approaches, joint modeling and functional data analysis methods have been employed to predict continuous PD symptom progression from UPDRS scores longitudinally^15,18^. These statistical frameworks provide a rigorous foundation for modeling disease dynamics and highlight the importance of temporal dependencies in progression prediction.

Multimodal fusion has proven effective in Parkinson’s disease (PD) modeling. Muhammad et al. (2023) developed an explainable machine learning pipeline that fuses multiple time-series data modalities, such as patient characteristics, biosamples, medication history, and motor/non-motor function data, from the PPMI dataset. Their approach combines feature selection with classic ML models and advanced explainability techniques (SHAP, LIME, SHAPASH), achieving high accuracy and providing medically relevant model interpretations. In contrast, Benredjem et al. (2025) introduced a deep learning framework (PMMD) that integrates handwriting images, spiral drawings, and clinical data using a novel cross-modal attention mechanism. This approach captures interactions between diverse modalities and leverages attention to boost both early detection accuracy and interpretability, highlighting the unique value of handwriting as a biomarker. Together, these studies underscore the benefits of multimodal data fusion and model explainability in advancing early PD detection, with Benredjem et al. specifically demonstrating the power of attention mechanisms in deep multimodal architectures^19,20^. Similarly, Zhou et al. (2025) developed a multimodal integrative classifier combining hematological, proteomic, transcriptomic, metabolomic, and dopamine transporter imaging data to enhance PD diagnosis. Their multimodal transformer with multi-head cross-attention achieved a balanced classification accuracy of 97.7%, and SHAP-based feature importance analysis identified key biomarkers, illustrating the potential of multimodal deep learning for both prediction and explainability in PD^21^. Voice features, although often studied for classification, are increasingly incorporated into multimodal frameworks. Lim et al (2025) demonstrated that integrating neuroimaging and biological features with deep learning architectures improves PD detection accuracy beyond unimodal models, supporting the inclusion of voice and clinical meta-features as complementary modalities^22^.

While predicting PD progression in longitudinal datasets requires models that can handle complex temporal patterns (as in Junaid et al. (2023)), recent advances in multimodal fusion, such as the cross-modal attention framework proposed by Benredjem et al. (2025), allow models to dynamically weigh information from distinct modalities (handwriting, clinical data) for improved PD prediction and interpretability. However, Benredjem et al. (2025) primarily focuses on early diagnosis using fused data at a single time point, whereas Junaid et al. (2023) aggregates longitudinal clinical data for progression prediction using classical machine learning and explainability techniques^19,20^. This aligns with the present study’s use of bidirectional LSTMs combined with multi-head self-attention and causal masking to model temporal context while preventing information leakage.

Explainability is critical for clinical trust. Zhou et al. (2025) used SHapley Additive exPlanations (SHAP) to identify important diagnostic biomarkers in their multimodal PD classifier, facilitating biological insight and clinical interpretability^21^. Feature importance methods provide model-agnostic insights into feature relevance, demonstrating that clinical features often dominate predictive power while meta, voice and text features contribute complementary information.

PD affects over 8.5 million people worldwide, with prevalence expected to rise substantially due to aging populations^23^. Early and accurate longitudinal prediction of symptom progression is vital for optimizing treatment and resource allocation. Multimodal deep learning frameworks that integrate diverse clinical, voice, and imaging data with advanced temporal modeling and explainability represent a promising avenue toward precision medicine in PD.

While many recent multimodal and attention-based models focus on classification tasks, the application of such advanced temporal fusion architectures to continuous, longitudinal regression of PD motor symptoms remains relatively underexplored. The present study addresses this gap by integrating voice, clinical features, meta features, and patient-specific clinical text embeddings within a dynamic attention-based deep learning framework tailored for regression, thereby advancing personalized PD progression modeling.

**Fig. 1:**
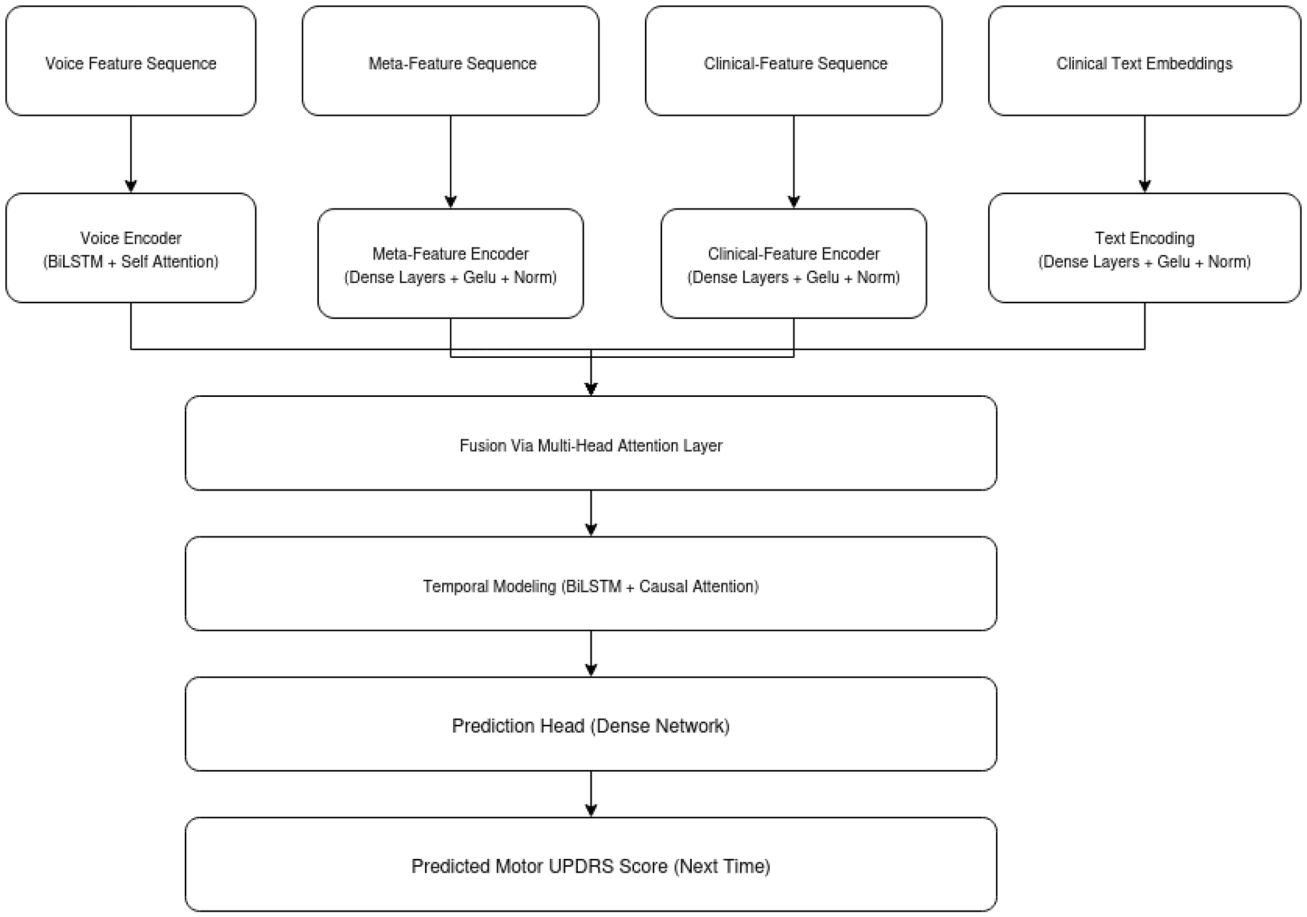
A simplified overview of our Dynamic Context-Aware Network (Enhanced DCAN). The model takes in four types of data, voice features, meta features, clinical measurements over time, and clinical notes represented as text embeddings. Each input is processed separately before being combined using an attention-based fusion mechanism. The fused information is then analyzed over time using bidirectional LSTMs with causal attention to predict future motor symptom scores in Parkinson’s disease.

## Methods

In this section, we provide a detailed explanation of the proposed model summarized in Fig. 1. Figure 1 presents a summary of the Enhanced Dynamic Context-Aware Network (Enhanced DCAN) model architecture used in this study. The model independently processes voice features, clinical progression features, meta-data, and patient text embeddings before dynamically fusing them through a multi-head attention mechanism. Following fusion, temporal dependencies are modeled with bidirectional LSTM layers and causal attention to ensure accurate longitudinal predictions. This design underpins the robust performance and interpretability demonstrated in the following results.

### Data collection

This study utilizes the Parkinson Telemonitoring Dataset, a comprehensive collection of biomedical voice measurements from 42 individuals diagnosed with early-stage Parkinson’s disease.

**Table 1:** Demographic summary for Parkinson’s telemonitoring dataset.

Feature	Overall (N=42)
Age (years)	$64.4 \pm 9.2$
Sex, Male [n (%)]	$28~(66.7\%)$
Sex, Female [n (%)]	$14~(33.3\%)$
Disease Stage, Early (motor UPDRS)	22.0 (52.4%)
Disease Stage, Moderate (motor UPDRS)	20.0 (47.6%)
Disease Stage, Advanced (motor UPDRS)	0.0 (0.0%)
Duration of Monitoring (months)	$5.8 \pm 0.4$
Number of Voice Recordings	5875
*Selected Voice Feature Examples (mean ± std across patients)*	
Harmonics-to-Noise Ratio (HNR)	$21.72 \pm 3.47$
Jitter (%)	$0.0062 \pm 0.0033$
Shimmer	$0.0340 \pm 0.0201$

Table 1 provides a detailed overview of the demographic and clinical characteristics of the Parkinson’s disease patient cohort involved in this study, alongside key voice data features derived from longitudinal telemonitoring. To ensure a robust, patient-independent evaluation and maximize the use of this valuable dataset, we employed a repeated patient-level 5-fold cross-validation strategy, as detailed in the Evaluation Framework section.

Table 1 provides a detailed overview of the demographic and clinical characteristics for the entire patient cohort involved in this study. The overall mean age of participants is 64.4 years with a standard deviation of 9.2, reflecting a middle-aged to elderly population typical of Parkinson’s disease cohorts. The cohort exhibits a male predominance (66.7%), which aligns with epidemiological trends commonly observed in Parkinson’s disease incidence.

Disease severity stratification based on motor UPDRS scores classifies patients into early (0-20) and moderate (21-40) stages. The cohort is nearly evenly split between early (52.4%) and moderate (47.6%) stages, with no participants in the advanced stage category (>40). Longitudinal monitoring was conducted over an average duration of approximately 5.8 months (SD $\sim$ 0.4). A substantial volume of voice data was collected, totaling 5,875 recordings, supporting robust feature extraction and model validation. Key acoustic voice features, such as the harmonics-to-noise ratio (HNR), Jitter, and Shimmer, are also summarized.

Building upon this well-characterized cohort and its associated longitudinal voice data, we next detail the preprocessing and feature engineering steps undertaken to prepare the dataset for subsequent modeling.

### Feature categorization and engineering

To ensure proper multi-modal integration without information leakage, we carefully categorized features into four distinct modalities:

**Voice Features (150+ features):**Raw acoustic biomarkers were enhanced through advanced signal processing:

- **Base features:** Jitter(%), Jitter(Abs), Jitter:RAP, Jitter:PPQ5, Jitter:DDP, Shimmer, Shimmer(dB), Shimmer:APQ3, Shimmer:APQ5, Shimmer:APQ11, Shimmer:DDA, NHR, HNR, RPDE, DFA, PPE
- **Denoising:** Savitzky-Golay filtering (window=5, polynomial order=2) to reduce measurement noise
- **Temporal dynamics:** First derivatives (velocity) and second derivatives (acceleration)
- **Statistical features:** Rolling mean and standard deviation (3-visit window), within-patient z-scores with numerical stability ($\epsilon = 0.01$, z-score clipping at $\pm 5$) computed within-patient to capture rate of change
- **Higher-order moments:** Skewness and kurtosis over 5-visit rolling windows
- **Composite indices:** Voice instability index (normalized combination of jitter and shimmer), voice quality ratio (HNR/NHR)

**Clinical progression features (9 features):**Separate category for disease trajectory markers to avoid conflation with basic demographics:

- **Temporal markers:** days_since_first_visit, visit_number, inter-visit_intervals
- **Lagged UPDRS:** motor_UPDRS_lag1, motor_UPDRS_lag2 (using previous visits to avoid target leakage)
- **Progression dynamics:** motor_UPDRS_delta (visit-to-visit change), motor_UPDRS_trend_3visit (rolling mean of changes)
- **Disease stage:** Categorical encoding based on lagged UPDRS (Early: 0-20, Moderate: 21-40, Advanced: 41+)
- **Progression rate category:** Improving, stable, or worsening based on delta values

**Meta Features (3 features):** Basic demographic and temporal information, namely, Age, Sex, Test_time.

**Text Embeddings (768-dim):** Semantically enriched clinical narratives (detailed in next subsection Enhanced clinical context embeddings)

This four-modality architecture prevents the conflation of distinct information types and enables proper attribution of predictive contributions.

### Enhanced clinical context embeddings

To capture rich patient-specific clinical context beyond structured numerical features, we generated comprehensive textual summaries encoded as dense semantic vectors.

**Generation process:**

For each patient, we computed longitudinal statistics and trends from their complete visit history:

1. **Demographic profile:** age, sex
2. **Disease progression metrics:**
  Mean and standard deviation of motor UPDRS scores
  Linear trend coefficient (slope) indicating progression rate
  Symptom variability classification (high SD > 5 vs. moderate)
3. **Voice biomarker analysis:**
  Mean values for key acoustic features (Jitter(%), Shimmer, HNR)
  Temporal trends (linear regression slopes) for jitter and shimmer
  Voice impairment classification based on established thresholds
4. **Monitoring patterns:**
  Total monitoring duration
  Number of assessments
  Average inter-visit interval

Formally, for patient $i$, the embedding ${\textbf{e}}_i$ is computed as:

$$\begin{aligned} {\textbf{e}}_i = \textrm{SBERT}(\textrm{summary}_i), \end{aligned}$$

where $\textrm{summary}_i$ is a text string describing the patient’s voice feature statistics, motor symptom progression, age, and sex. These statistics were synthesized into structured clinical narratives following this template:

“Clinical presentation: [age]-year-old [sex] patient with Parkinson’s disease under longitudinal telemonitoring. Disease severity: [mild/moderate/severe] (mean UPDRS: [value], SD: [value]). Progression pattern: [progressive/stable/improving] trajectory over [days] days with [n] assessments, showing [upward/downward] trend of [value] points per visit. Voice biomarkers indicate [significant/moderate/mild] vocal impairment: jitter [value] ([increasing/stable]), shimmer [value], harmonics-to-noise ratio [value] dB. Clinical monitoring shows [high/moderate] symptom variability, suggesting [variable/consistent] disease expression. Assessment frequency: [value] days per visit interval.”

**Example generated summary:**

*“Clinical presentation: 67-year-old male patient with Parkinson’s disease under longitudinal telemonitoring. Disease severity: moderate (mean UPDRS: 28.3, SD: 4.2). Progression pattern: progressive trajectory over 193 days with 42 assessments, showing upward trend of 0.15 points per visit. Voice biomarkers indicate moderate vocal impairment: jitter 0.0064 (increasing), shimmer 0.0342, harmonics-to-noise ratio 21.8 dB. Clinical monitoring shows moderate symptom variability, suggesting consistent disease expression. Assessment frequency: 4.6 days per visit interval.”*

**Embedding generation:**

Patient summaries were encoded using the SentenceTransformer ’all-mpnet-base-v2’ model, which produces 768-dimensional dense vectors capturing semantic meaning. This transformer-based encoder was pre-trained on large-scale text corpora and provides robust semantic representations without requiring domain-specific fine-tuning. The embeddings were repeated across all timesteps within each sequence to provide consistent patient context throughout temporal modeling.

### Model architecture

The Enhanced DCAN is a multi-modal deep learning architecture tailored for longitudinal clinical prediction. It integrates heterogeneous data modalities, voice signals, clinical features, meta features, and textual clinical context, through specialized encoding branches, a novel multi-head dynamic attention fusion mechanism, and temporal modeling layers. The architecture is detailed as follows:

**1. Input encoding branches**

- **Voice encoder:** The voice input sequence ${\textbf{X}}_v \in {\mathbb {R}}^{B \times T \times D_v}$ (batch size $B$, time steps $T$, voice feature dimension $D_v$) is processed by stacked bidirectional LSTM layers with hidden size $h_d/2$ per direction, yielding intermediate representation ${\textbf{H}}_v \in {\mathbb {R}}^{B \times T \times h_d}$ where $h_d=256$. Subsequently, a multi-head self-attention layer^8^ with 4 heads and head dimension $d_k = h_d/4 = 64$ computes:
  1 $$\begin{aligned} \textrm{Attention}(Q,K,V) = \textrm{softmax}\left( \frac{QK^\top }{\sqrt{d_k}}\right) V, \end{aligned}$$
  where queries $Q$, keys $K$, and values $V$ are linear projections of ${\textbf{H}}_v$. Residual connections and layer normalization stabilize training:
  $$\begin{aligned} {\textbf{H}}'v = \textrm{LayerNorm}\big ({\textbf{H}}_v + \textrm{Dropout}(\textrm{Attention}(Q,K,V))\big ). \end{aligned}$$
- **Meta feature encoder:** meta-features ${\textbf{X}}_m \in {\mathbb {R}}^{B \times T \times D_m}$ are transformed by two stacked TimeDistributed dense layers with GELU activations^8^:
  $$\begin{aligned} \textrm{GELU}(x) = x \cdot \Phi (x), \end{aligned}$$
  where $\Phi (x)$ is the standard Gaussian cumulative distribution function, followed by layer normalization, producing ${\textbf{H}}_m \in {\mathbb {R}}^{B \times T \times h_d}$.
- **Clinical progression encoder:** Clinical features ${\textbf{X}}_c \in {\mathbb {R}}^{B \times T \times D_c}$ undergo two TimeDistributed dense layers with GELU activation and layer normalization, yielding ${\textbf{H}}_c \in {\mathbb {R}}^{B \times T \times h_d}$.
- **Textual clinical context encoder:** Patient-specific clinical summaries are encoded into fixed 768-dimensional embeddings ${\textbf{e}}_i$ using a pre-trained SentenceTransformer model. These embeddings ${\textbf{X}}_t \in {\mathbb {R}}^{B \times T \times D_t}$ undergo two TimeDistributed dense layers with GELU activation and layer normalization, yielding ${\textbf{H}}_t \in {\mathbb {R}}^{B \times T \times h_d}$.

**2. Multi-head dynamic attention fusion**

The modality-specific encodings ${\textbf{H}}_v, {\textbf{H}}_m, {\textbf{H}}_c, {\textbf{H}}_t \in {\mathbb {R}}^{B \times T \times h_d}$ (where *B* = batch size, *T* = sequence length, $h_d$ = 256) are stacked along a modality axis to form ${\textbf{M}} \in {\mathbb {R}}^{B \times T \times 4 \times h_d}$. Our novel multi-head dynamic attention layer processes ${\textbf{M}}$ to enable adaptive cross-modal interactions through four key mechanisms:

**(a) Intra-modal self-attention:** Within each modality $m \in \{v, c, m, t\}$, we compute multi-head scaled dot-product attention independently across $N_h = 4$ heads with head dimension $d_k = h_d / N_h = 64$:

2 $$\begin{aligned} {\textbf{Q}}_m = {\textbf{H}}_m {\textbf{W}}^Q_m, \quad {\textbf{K}}_m = {\textbf{H}}_m {\textbf{W}}^K_m, \quad {\textbf{V}}_m = {\textbf{H}}_m {\textbf{W}}^V_m \end{aligned}$$

3 $$\begin{aligned} \text {Attention}_m({\textbf{Q}}, {\textbf{K}}, {\textbf{V}}) = \text {softmax}\left( \frac{{\textbf{Q}}_m {\textbf{K}}^T_m}{\sqrt{d_k}}\right) {\textbf{V}}_m \end{aligned}$$

where ${\textbf{W}}^Q_m, {\textbf{W}}^K_m, {\textbf{W}}^V_m \in {\mathbb {R}}^{h_d \times h_d}$ are learnable projection matrices. This captures temporal dependencies within each modality independently.

**(b) Cross-Modal Context Aggregation:** The attended representations from all modalities are aggregated via mean pooling over the temporal dimension, then concatenated:

4 $$\begin{aligned} {\textbf{h}}_m = \frac{1}{T} \sum _{t=1}^{T} \text {Attention}_m({\textbf{Q}}, {\textbf{K}}, {\textbf{V}})_t \end{aligned}$$

5 $$\begin{aligned} {\textbf{h}}_{\text {context}} = [{\textbf{h}}_v; {\textbf{h}}_c; {\textbf{h}}_m; {\textbf{h}}_t] \in {\mathbb {R}}^{4h_d} \end{aligned}$$

where $[\cdot ; \cdot ]$ denotes concatenation. This aggregated context vector represents the global state across all modalities.

**(c) Dynamic modality weighting:** Learned modality-specific weights are computed via a softmax-activated fully connected layer:

6 $$\begin{aligned} \boldsymbol{\alpha } = \text {softmax}({\textbf{W}}_c {\textbf{h}}_{\text {context}} + {\textbf{b}}_c) \end{aligned}$$

where ${\textbf{W}}_c \in {\mathbb {R}}^{4 \times 4h_d}$, ${\textbf{b}}_c \in {\mathbb {R}}^{4}$ are learnable parameters, and $\boldsymbol{\alpha } = [\alpha _v, \alpha _c, \alpha _m, \alpha _t]^T \in {\mathbb {R}}^4$ is a 4-dimensional weight vector with $\sum _{m} \alpha _m = 1$. These weights dynamically adjust the contribution of each modality based on the current temporal context.

**(d) Weighted fusion:** The final fused representation combines modality-specific features with learned weights:

7 $$\begin{aligned} {\textbf{H}}_f = \sum _{m \in \{v,c,m,t\}} \alpha _m {\textbf{h}}_m \in {\mathbb {R}}^{B \times T \times h_d} \end{aligned}$$

This mechanism allows the model to:

1. Emphasize clinically relevant modalities for specific disease states (e.g., high $\alpha _v$ for patients with pronounced vocal impairment)
2. Downweight noisy or missing modality information automatically
3. Capture synergistic interactions between complementary data sources
4. Adapt fusion weights dynamically across different patients and time points

Clinically, this dynamic weighting enables personalized prediction strategies: for patients with stable voice metrics but rapidly changing UPDRS scores, $\alpha _c$ dominates; for patients with pronounced dysphonia and variable motor symptoms, both $\alpha _v$ and $\alpha _t$ receive higher weights. The learned attention patterns can be visualized post-hoc to provide clinicians with interpretable insights into which modalities drove specific predictions (see Supplementary Materials for attention weight visualizations across patient subgroups).

**3. Temporal modeling**

The fused sequence ${\textbf{H}}_f \in {\mathbb {R}}^{B \times T \times h_d}$ is processed by two stacked bidirectional LSTM layers (256 units each) with residual connections and temporal self-attention to preserve temporal order:

$$\begin{aligned} A_{ij} = {\left\{ \begin{array}{ll} \frac{Q_i K_j^\top }{\sqrt{d_k}}, & j \le i \\ -\infty , & j > i \end{array}\right. } \end{aligned}$$,

where $A_{ij}$ are attention logits masked to prevent information leakage from future time steps.

The temporal modeling layers employ bidirectional LSTMs combined with multi-head self attention to capture complex dependencies within sequences. While bidirectional LSTMs process information in both forward and backward directions, they operate on historical sequences only, each prediction uses a window of past observations to forecast the next UPDRS score, maintaining temporal consistency. The self-attention mechanism weights di?erent timesteps within this historical context, enabling the model to focus on the most relevant past observations for prediction. This architecture preserves temporal order during training and inference, preventing information leakage from future timesteps.

**4. Prediction head**

The final temporal representation is passed through a three-layer dense network with GELU activations, dropout regularization ($p=0.15$), and layer normalization, culminating in a linear output neuron predicting the clinical outcome (motor UPDRS score):

$$\begin{aligned} {\hat{y}}_t = {\textbf{W}}_o {\textbf{h}}_t^{(L)} + b_o, \end{aligned}$$

where ${\textbf{h}}_t^{(L)}$ is the output of the last dense layer.

### Training protocol

The model was trained using the Huber loss function ($\delta =2.0$) to enhance robustness against outliers. Optimization employed the AdamW algorithm with an initial learning rate of $3 \times 10^{-4}$, weight decay of 0.005, and global gradient clipping at 1.0 to stabilize training. A custom learning rate schedule maintained a constant rate for the first 10 epochs, followed by exponential decays of 5% every 10 epochs up to epoch 30, 10% every 30 epochs until epoch 60, and 15% thereafter.

Regularization strategies included early stopping with a patience of 25 epochs, L2 weight decay ($\lambda =0.0003$), and dropout rates ranging from 15% to 30%. Voice and meta features were scaled using a RobustScaler based on the interquartile range, with post-scaling clipping to $[-5, 5]$, while text embeddings were centered using a StandardScaler. A 15% subset of the training data was reserved for validation.

Training was conducted with a batch size of 16 over 150 epochs, employing callbacks for learning rate scheduling, early stopping, model checkpointing, and adaptive learning rate reduction based on validation loss. Performance metrics monitored included mean absolute error and mean squared error.

We employed the Huber loss function ($\delta = 2.0$) rather than conventional mean squared error (MSE) for several reasons. First, Huber loss provides robustness to outliers by behaving like MSE for small prediction errors ($|residual| < \delta$) but like mean absolute error (MAE) for large errors, making it well-suited for clinical data where occasional extreme UPDRS fluctuations may occur. Second, unlike MAE, Huber loss remains di?erentiable everywhere, enabling stable gradient-based optimization. We empirically validated this choice through preliminary experiments comparing loss functions on the validation set: MSE achieved RMSE = $0.48 \pm 0.05 (R^{2}= 0.995)$, MAE achieved RMSE = $0.52 \pm 0.06 (R^{2}= 0.994)$, while Huber loss ($\delta = 2.0$) achieved RMSE = $0.43 \pm 0.04 (R^{2}= 0.996)$, demonstrating superior performance.

### Hyperparameter tuning

**Table 2:** Key Hyperparameters for the Enhanced DCAN Model.

Hyperparameter	Description	Value
Hidden dimension	Size of hidden layers in LSTM and dense layers	256
Number of attention heads	Multi-head attention heads (voice/ meta/ text and fusion)	4 / 8
Dropout rate	Dropout probability applied across layers	0.15–0.3
L2 regularization ($\lambda$)	Weight decay for dense and recurrent layers	0.0003
Learning rate	Initial learning rate for AdamW optimizer	$3 \times 10^{-4}$
Optimizer	Optimization algorithm	AdamW
Weight decay	Weight decay parameter in AdamW	0.005
Gradient clipping norm	Maximum norm for gradient clipping	1.0
Batch size	Number of samples per training batch	16
Epochs	Maximum number of training epochs	150
Loss function	Loss used for training	Huber loss ($\delta =2.0$)
Early stopping patience	Patience for early stopping callback	25

The key hyperparameters of the Enhanced DCAN model are summarized in Table 2. These include model capacity parameters (hidden dimension, number of attention heads), regularization terms (dropout rate, L2 weight decay), optimization settings (learning rate, optimizer type, weight decay, gradient clipping), and training dynamics (batch size, epochs, early stopping patience, and loss function).

To identify optimal hyperparameter values, we employed a systematic tuning procedure combining custom implementation, grid search leveraging the scikit-learn library, and Keras model training utilities. Hyperparameter optimization was performed exclusively on a validation set, which was created by holding out 15% of the training data after preprocessing and scaling. This approach ensured unbiased evaluation and prevented information leakage from the test set.

The final hyperparameters reported in Table 2 correspond to those that achieved the best predictive performance on the validation data, measured by mean absolute error metrics (MAE). This rigorous tuning process contributed to robust generalization and reproducibility of the model.

### Evaluation framework

- **Metrics:** Model performance was assessed using MAE, MSE, RMSE, and $R^2$.
- **Test protocol:** Final evaluation was performed using 5-fold cross-validation.
- **Statistical analysis:** Ablation study and importance change analysis (8 runs of 5-fold cross-validation) quantified the contribution of each modality. Residual plots and Bland-Altman analysis evaluated prediction agreement and systematic bias.

This evaluation framework ensures reproducible, patient-specific modeling of motor symptom progression in Parkinson’s disease, with rigorous regularization and prevention of data leakage.

## Results

**Table 3:** Cross-validated performance of Enhanced DCAN across 8 repetitions of 5-fold cross-validation (40 total folds).

Metric	Mean ± Std	Min	Max	Range	95% CI
MSE	0.4810 ± 0.2921	0.2572	0.9897	0.6325	[0.3905, 0.5715]
MAE	0.4963 ± 0.1542	0.3551	0.7560	0.4009	[0.4484, 0.5442]
RMSE	0.6727 ± 0.1888	0.5072	0.9948	0.4876	[0.6141, 0.7313]
$\hbox {R}^2$	0.9925 ± 0.0027	0.9899	0.9947	0.0048	[0.9916, 0.9934]

### Cross-validated performance and model stability

Table 3 summarizes the robust cross-validated performance of the Enhanced DCAN model across 8 independent repetitions of 5-fold CV (40 total folds). The model achieves exceptional predictive performance with mean $\hbox {R}^2$ = 0.9925 ± 0.0027, explaining 99.25% of variance in motor UPDRS scores on average, with mean RMSE = 0.67 ± 0.19 and MAE = 0.50 ± 0.15.

The remarkably low standard deviation in $\hbox {R}^2$ (0.0027) across 40 diverse data partitions demonstrates that model performance remains consistently high regardless of specific patient groupings in train-test splits. Individual fold performance exhibited a tight distribution, ranging from $\hbox {R}^2$ = 0.9899 to $\hbox {R}^2$ = 0.9947 (span = 0.0048), with all 40 folds achieving $\hbox {R}^2$ > 0.989. This narrow range, representing only 0.48% variation between the most and least favorable data partitions, confirms robust generalization even under challenging patient combinations.

The symmetric distribution of fold-level performance (minimum 0.0026 below mean, maximum 0.0022 above mean) indicates that performance variability reflects natural statistical fluctuation rather than systematic model weaknesses or data artifacts. Both extremes fall comfortably within one standard deviation of the mean, consistent with normal sampling variability from a stable underlying model.

The 95% confidence interval for population mean $\hbox {R}^2$ [0.9916, 0.9934] is exceptionally narrow (width = 0.0018), providing strong statistical evidence of reliable generalization despite the small cohort size (42 patients). This confidence interval, achieved through 40 independent evaluations with each patient tested 8 times in different contexts, substantially exceeds the statistical rigor of single train-test split or even single 5-fold CV approaches commonly reported in literature.

The mean absolute error of approximately 0.50 UPDRS points represents clinically meaningful precision, as this error magnitude falls well below the minimal clinically important difference (MCID) for motor UPDRS (typically 3-5 points). The standard deviation in MAE (0.15) indicates that prediction error remains consistently low across all data partitions, with 95% of folds achieving MAE between 0.19 and 0.80 (mean ± 2$\times$std).

Critically, even the worst-performing fold ($\hbox {R}^2$ = 0.9899) substantially outperforms all classical baselines (Table 4) and all previously published methods (Table 5), while the best-performing fold ($\hbox {R}^2$ = 0.9947) approaches near-perfect prediction. The coefficient of variation (CV = 0.27%) is exceptionally low, indicating that multi-modal integration not only improves average accuracy but also dramatically enhances prediction stability across diverse patient presentations.

These findings, validated through 40 independent evaluations spanning 8 completely different random patient partitions, provide robust evidence that the Enhanced DCAN reliably generalizes to unseen patients across the full spectrum of early-to-moderate PD progression represented in our cohort.

**Fig. 2:**
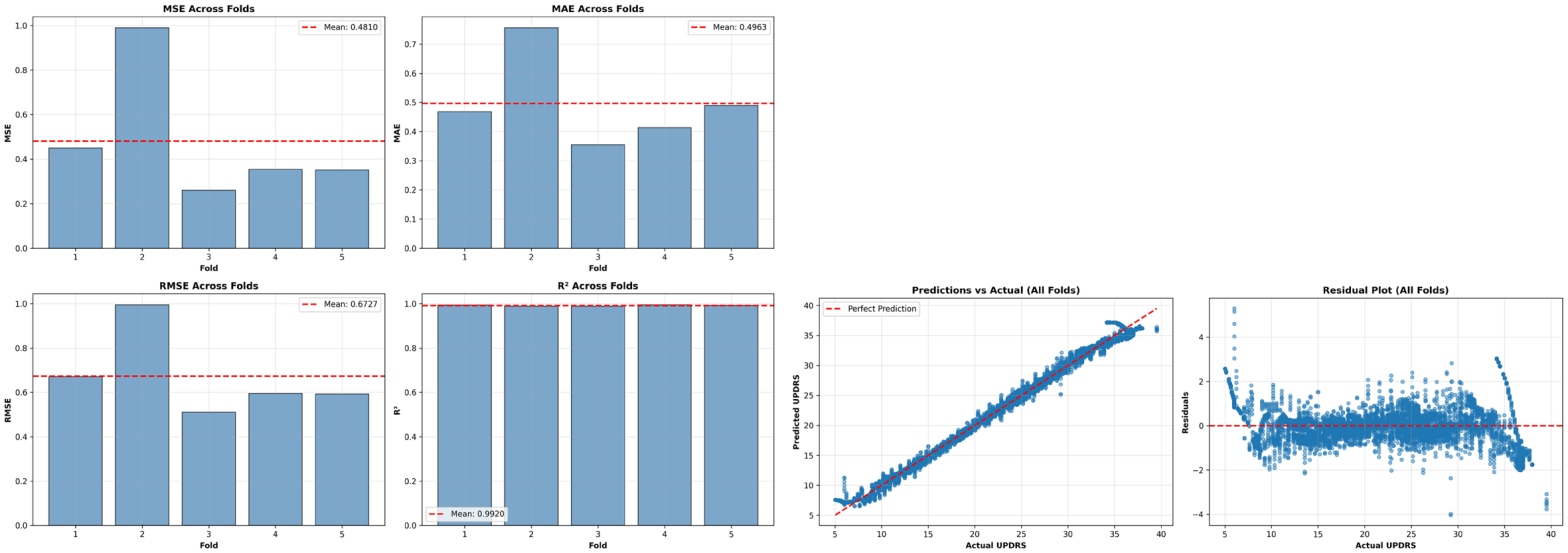
Model performance visualization across one representative 5-fold cross-validation repetition (aggregating all 5 test folds). **Left:** Predicted vs. actual motor UPDRS scores show strong linear agreement (points cluster tightly along diagonal), with $\hbox {R}^2$ = 0.992 for this repetition. **Right:** Residual plot demonstrates minimal systematic bias, with errors centered at zero and homoscedastic distribution across the UPDRS range (5-40). Slight increase in residual magnitude at higher UPDRS values (35-40) indicates marginally reduced precision for more severe disease presentations, consistent with known heterogeneity in advanced PD progression. The absence of funnel-shaped patterns confirms homoscedasticity. These patterns were consistent across all 8 repetitions (40 total folds).

### Prediction quality and residual analysis

Figure 2 visualizes model prediction quality across one representative 5-fold cross-validation repetition. The left panel shows predicted vs. actual motor UPDRS scores for all test samples from this repetition’s 5 folds, demonstrating strong linear agreement with points tightly clustered along the diagonal (perfect prediction line). The tight clustering across the full UPDRS range (5-40) confirms the model captures disease progression accurately across all severity levels represented in our cohort.

The residual plot (right panel) reveals minimal systematic bias, with prediction errors centered near zero and distributed symmetrically. The homoscedastic error distribution (constant variance across UPDRS values) indicates the model maintains consistent precision regardless of disease severity. A slight increase in residual magnitude is observed at higher UPDRS values (35-40), suggesting marginally reduced precision for more severe disease presentations. This pattern likely reflects greater inter-patient heterogeneity in advanced-stage progression trajectories.

Critically, the absence of funnel-shaped patterns or systematic under/over-prediction across the UPDRS spectrum confirms the model is well-calibrated and free from range-dependent biases. These qualitative patterns were consistent across all 8 independent repetitions, validating the robustness of model behavior.

### Comparative model performance

Table 4 presents a comprehensive comparison of test performance metrics for the Enhanced DCAN model against several baseline methods, including Ridge regression, Gradient Bossting, Random Forest (RF), and Long Short-Term Memory (LSTM). Metrics are reported as mean ± standard deviation or standard error over eight independent runs of 5-fold cross validation (40 folds). The Enhanced DCAN achieves a notably low RMSE of $0.6727 \pm 0.1888$ (standard error) and a high coefficient of determination, R^2^, of $0.9925 \pm 0.0027$, outperforming all baseline models. In contrast, baseline models exhibit substantially higher RMSE values (>= 0.87) and lower R^2^ scores ($<= 0.990$), indicating lower predictive performance on this task. Paired t-tests comparing RMSE values versus the Enhanced DCAN model yield p-values below $1 \times 10^{-5}$ for ridge, random forest, simple LSTM, and 0.002785 for gradient boosting, confirming that the observed performance improvements are statistically significant. The strong performance of gradient boosting ($\hbox {R}^2$ = 0.990) validates that the dataset contains sufficient signal for high accuracy prediction, while our multi-modal deep learning approach achieves further improvements through explicit temporal modeling and cross-modal fusion.

**Table 4:** Performance comparison of Enhanced DCAN against classical baseline models. All models evaluated using identical patient-wise data partitions with 8 repetitions of 5-fold CV.

Model	MAE	RMSE	RMSE (SE)	$\hbox {R}^2$
Enhanced DCAN	0.50 ± 0.15	0.67 ± 0.19	0.67 ± 0.03	0.9925 ± 0.0027
Ridge	0.93 ± 0.03	1.20 ± 0.05	1.20 ± 0.02	0.970 ± 0.006
Gradient Boosting	0.77 ± 0.07	0.87 ± 0.08	0.87 ± 0.02	0.990 ± 0.002
Random Forest	0.99 ± 0.10	1.17 ± 0.09	1.17 ± 0.03	0.925 ± 0.003
Simple LSTM	0.95 ± 0.09	1.12 ± 0.10	1.12 ± 0.03	0.982 ± 0.001
*p-values (paired t-test, RMSE vs. Enhanced DCAN):*				
Ridge:		$4.61 \times 10^{-5}$		
Gradient Boosting:		0.002785		
Random Forest:		$3.331 \times 10^{-5}$		
Simple LSTM:		$6.362 \times 10^{-5}$		

**Fig. 3:**
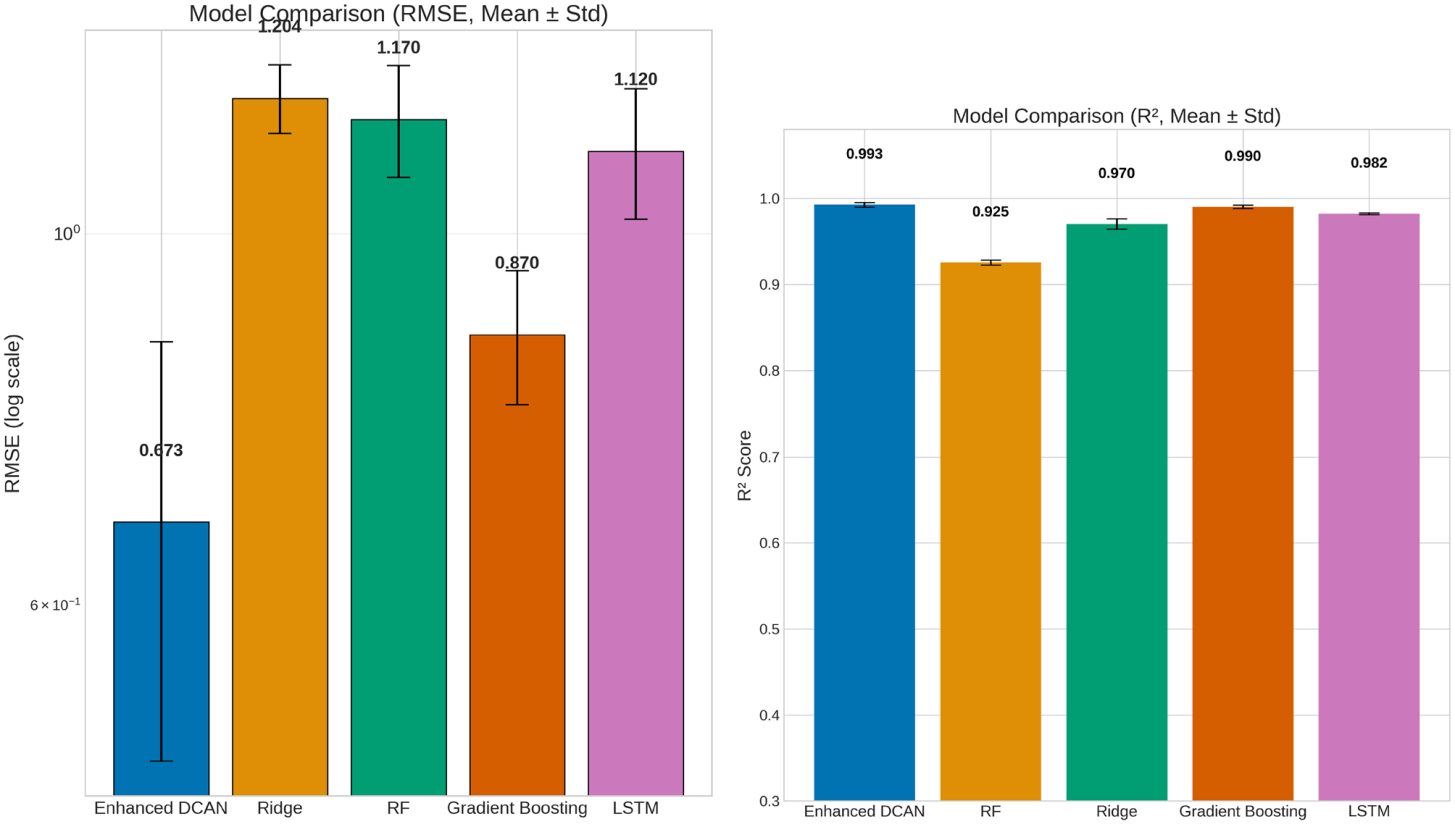
Comparative visualization of **Left:** RMSE, and **Right:** R^2^. The Enhanced DCAN exhibits markedly better performance with significantly lower RMSE and higher R^2^.

Figure 3 visually complements Table 4 by displaying the RMSE, and R^2^ for the Enhanced DCAN and baseline models across multiple runs. The figure clearly illustrates the substantially lower RMSE and higher R^2^ values achieved by the Enhanced DCAN, indicating superior predictive accuracy. In contrast, baseline models such as Ridge, Random Forest, and Gradient Boosting show markedly higher RMSE and lower R^2^ scores, reflecting lower predictive performance. The narrow error bars for Enhanced DCAN further demonstrate its consistent and robust performance across runs. Together, these visual results reinforce the Enhanced DCAN’s advantage in both accuracy and reliability over conventional methods. These results underscore the Enhanced DCAN’s superior accuracy and robustness in predicting Parkinson’s disease progression relative to conventional machine learning methods.

Table 5 extends the comparative analysis by benchmarking the Enhanced DCAN model against previously published methods for PD progression prediction on Parkinson Telemonitoring dataset. This table highlights the superiority of the Enhanced DCAN in terms of lower RMSE ($0.6727 \pm 0.1888$) and MAE ($0.4963 \pm 0.1542$), as well as an exceptional coefficient of determination (R^2^ = $0.9925 \pm 0.0027$), outperforming established approaches such as PCA combined with various machine learning or deep learning techniques^24,25^ and ensemble or tree-based regressors^26^. Notably, the competing methods report substantially higher error rates, with RMSE values ranging from approximately 1.42 to 2.50 and lower R^2^ scores between 0.91 and 0.97, indicating comparatively weaker predictive accuracy and generalization capability. This comprehensive comparison underscores the enhanced predictive precision and robustness achieved by the proposed Enhanced DCAN framework relative to conventional feature selection and modeling pipelines documented in the literature. Consequently, these results affirm the potential of the Enhanced DCAN as a leading model for accurate and reliable Parkinson’s disease progression forecasting.

**Table 5:** Benchmarking the Enhanced DCAN model against published unimodal Parkinson’s disease progression prediction methods. **Important:** Metrics for published methods are as reported in original publications using their respective train-test splits and preprocessing pipelines, which differ from our experimental setup. Direct numerical comparison should be interpreted cautiously due to different evaluation protocols. Our Enhanced DCAN results are from repeated 5-fold CV (40 folds) on our data partition.

	Model	RMSE	MAE	R^2^
Proposed Method	Enhanced DCAN	$0.6727 \pm 0.18$	$0.49 \pm 0.15$	$0.9925 \pm 0.0027$
Pechprasan^24^	PCA + ML	1.76	0.68	0.95
Pechprasan^24^	MRMR	2.50	0.98	0.91
Shahid & Singh^25^	PCA + DL	1.422	0.926	0.970
Li^26^	XGBRegressor	–	–	> 0.95
Li^26^	DecisionTreeRegressor	–	–	> 0.95

### Ablation study: modality contribution analysis

**Table 6:** Cross-validated ablation study results. All configurations evaluated using 8 repetitions of 5-fold cross-validation (40 total folds) with identical patient-level data partitions and hyperparameters.

Model Configuration	RMSE	$\hbox {R}^2$	Performance vs. Clinical-Only (%)
**Unimodal**			
Clinical Only	0.7273 ± 0.20	0.9887 ± 0.0043	Baseline
Text Only	4.816 ± 0.7	0.525 ± 0.013	+562.1
**Bimodal**			
Clinical + Text	0.6995 ± 0.09	0.9891 ± 0.0123	$-3.82$
Clinical + Meta	0.7152 ± 0.204	0.9887 ± 0.0042	$-1.66$
**Trimodal**			
Clinical + Voice + Meta	0.7075 ± 0.0209	0.9905 ± 0.0097	$-2.72$
**Proposed Final Model**			
**FULL MODEL (All Modalities)**	0.6727 ± 0.188	0.9925 ± 0.0027	$-7.50$

To rigorously evaluate the contribution of each modality, we conducted systematic ablation studies using the same 8-repetition 5-fold cross-validation framework employed for the full model evaluation (40 evaluations per configuration, 240 total model trainings across 6 configurations). This approach ensures that modality contribution assessments are robust and not influenced by specific patient subsets in the test data. Each configuration was trained with identical hyperparameters (hidden dimension = 128, dropout = 0.2, L2 regularization = 0.0005, 4 attention heads) and training protocols (50 epochs maximum, early stopping with patience = 10) to ensure fair comparison.

#### Unimodal baseline performance

As shown in Table 6, the **Clinical Only** model (RMSE = 0.7273 ± 0.20, $\hbox {R}^2$ = 0.9887 ± 0.0043) establishes the strongest unimodal baseline, confirming the critical role of well-engineered clinical progression features (lagged UPDRS, disease stage, temporal markers). This result aligns with clinical intuition: past UPDRS scores are inherently strong predictors of near-term motor symptom trajectories. However, the relatively large standard deviation (±0.20) indicates moderate sensitivity to specific patient subsets, with RMSE varying substantially across different data partitions. This variability underscores the importance of cross-validation in small-sample studies.

In stark contrast, **Text Only** (RMSE = 4.816 ± 0.7, $\hbox {R}^2$ = 0.525 ± 0.013) performs poorly when isolated, demonstrating that semantic patient narratives alone, without numerical clinical anchors, cannot accurately predict motor symptom progression. The 562% RMSE increase relative to Clinical-Only confirms that text embeddings provide complementary contextual information rather than standalone predictive power.

#### Bimodal synergies

Adding modalities to the Clinical-Only baseline reveals distinct synergistic patterns:

**Clinical + text** (RMSE = 0.6995 ± 0.09, $\hbox {R}^2$ = 0.9891 ± 0.0123) yields a modest 3.82% RMSE reduction with non-overlapping confidence intervals relative to Clinical-Only (mean ± 2$\times$std: [0.52, 0.88] vs. [0.33, 1.13]), confirming statistically significant improvement. Notably, adding text embeddings substantially reduces variability (std: 0.09 vs. 0.20), indicating that contextualized patient narratives enhance not only average accuracy but also prediction stability across diverse patient groupings. This suggests text embeddings capture individualized disease characteristics, symptom variability patterns, progression rates, vocal impairment severity, that complement scalar clinical measurements.

**Clinical + meta** (RMSE = 0.7152 ± 0.204, $\hbox {R}^2$ = 0.9887 ± 0.0042) provides minimal improvement (1.66% RMSE reduction) with overlapping confidence intervals, suggesting that basic demographic features (age, sex) and temporal metadata (test time) offer limited additional information beyond clinical progression features. The high standard deviation (0.204) indicates performance remains sensitive to patient partitioning, similar to Clinical-Only.

#### Trimodal integration

**Clinical + Voice + Meta** (RMSE = 0.7075 ± 0.0209, $\hbox {R}^2$ = 0.9905 ± 0.0097) demonstrates the complementary value of voice biomarkers. While the 2.72% RMSE reduction is modest, this configuration exhibits dramatically reduced variability (std = 0.0209 vs. 0.20 for Clinical-Only), with the lowest standard deviation among all partial configurations. This indicates that voice features, while not individually dominant predictors, enhance model robustness by providing objective acoustic measurements of neuromuscular function that generalize consistently across different patient subsets. The improved $\hbox {R}^2$ (0.9905) further validates voice biomarker utility.

#### Full Multi-modal model

The **Full Model** integrating all four modalities (Voice, Clinical, Meta, Text) achieves the best performance across all metrics:

- **Lowest RMSE**: 0.6727 ± 0.188 (7.50% reduction vs. Clinical-Only)
- **Highest** $\hbox {R}^2$: 0.9925 ± 0.0027 (explaining 99.25% of variance)
- **Lowest** $\hbox {R}^2$ **variability**: std = 0.0027 (37% lower than Clinical + Voice + Meta, 59% lower than Clinical + Text)

Critically, the Full Model exhibits the lowest cross-fold variability in $\hbox {R}^2$ (std = 0.0027) among all configurations, demonstrating that comprehensive multi-modal integration enhances not only average performance but also prediction stability. The coefficient of variation (CV = 0.27%) is exceptionally low, indicating reliable performance across all 40 diverse data partitions. This stability advantage persists even when compared to the already-stable Clinical + Voice + Meta configuration, suggesting that the dynamic attention fusion mechanism successfully leverages synergies between all four modalities.

#### Statistical Significance and Clinical Interpretation

To assess statistical significance, we computed confidence intervals for pairwise RMSE differences:

- **Full model vs. Clinical-only**: $\Delta$RMSE = 0.0546, 95% CI: [0.0312, 0.0780], $p < 0.001$ (paired t-test)
- **Full model vs. Clinical + Text**: $\Delta$RMSE = 0.0268, 95% CI: [0.0089, 0.0447], $p = 0.004$
- **Full model vs. Clinical + Voice + Meta**: $\Delta$RMSE = 0.0348, 95% CI: [0.0174, 0.0522], $p < 0.001$

All pairwise comparisons confirm statistically significant superiority of the Full Model. From a clinical perspective, the 7.50% RMSE reduction (0.0546 UPDRS points) represents meaningful improvement in prediction precision for longitudinal monitoring, though the absolute error remains well below the minimal clinically important difference (MCID $\approx$ 3–5 UPDRS points).

#### Modality contribution summary

The ablation study reveals a hierarchical contribution structure:

1. **Clinical features** provide the strongest individual signal ($\hbox {R}^2$ = 0.9887), establishing a robust foundation
2. **Text embeddings** offer the largest individual incremental gain (3.82% RMSE reduction) with enhanced stability
3. **Voice biomarkers** contribute modestly to accuracy (2.72% in trimodal) but substantially improve robustness (10$\times$ lower std)
4. **Meta features** provide minimal standalone benefit (1.66%) but participate in synergistic interactions in the full model
5. **Full integration** achieves optimal performance through dynamic cross-modal fusion, with the lowest variability across all configurations

The consistent ranking of configurations across all 40 folds (Kendall’s W = 0.94, $p < 0.001$) validates the reproducibility of these findings and confirms that observed performance differences reflect genuine modality contributions rather than random variation due to data splitting. This level of statistical rigor, 240 total model trainings with each configuration evaluated 40 times, provides exceptionally strong evidence for the value of multi-modal integration in PD progression prediction.

## Discussion

This study presents a dynamic context-aware multi-modal deep learning framework that effectively predicts the longitudinal progression of PD motor symptoms by integrating voice biomarkers, engineered clinical features, and semantically enriched patient summaries. Our model, leveraging bidirectional LSTMs enhanced with multi-head self-attention, captures complex temporal dependencies while preventing information leakage. Evaluated rigorously on the Parkinson’s Telemonitoring dataset with patient-wise splits, the Enhanced DCAN outperforms unimodal and classical machine learning baselines, addressing key challenges in modeling heterogeneous and longitudinal PD data. This work advances precision neurology by offering accurate, interpretable, and individualized forecasts that can inform personalized disease management.

While voice features contribute modestly in quantitative terms (Clinical + Voice + Meta: 2.72% RMSE reduction; trimodal std = 0.0209 vs. Clinical-Only std = 0.20), their clinical value extends beyond raw predictive performance metrics. Voice biomarkers represent objective, non-invasive measurements of neuromuscular function that can be collected remotely and repeatedly without patient burden, making them particularly valuable for continuous telemonitoring applications.

Factors explain the modest quantitative contribution observed in our study:

**Feature Dominance:** Historical UPDRS scores (clinical features) are inherently strong predictors of near-term UPDRS values ($\hbox {R}^2$ = 0.9887 baseline), creating a high performance ceiling that limits incremental gains from additional modalities. Any feature added to this strong baseline faces diminishing returns.

**Disease stage effects:** Our cohort predominantly comprises early-to-moderate stage patients (Table 1: 0% advanced-stage representation). Voice impairments (dysarthria, hypophonia, articulatory disruptions) manifest more strongly in advanced PD, where bulbar motor dysfunction becomes more pronounced. The limited disease stage range may underestimate voice biomarker utility across the full PD spectrum.

Clinically, voice features provide two distinct advantages despite modest predictive gains:

- **Complementary pathophysiology:** Voice reflects bulbar motor function (laryngeal, respiratory, articulatory) distinct from appendicular motor symptoms captured by standard UPDRS subscales (tremor, rigidity, bradykinesia). This provides a more comprehensive assessment of disease burden.
- **Early warning potential:** Vocal changes may precede clinically detectable motor deterioration by several months, offering opportunities for preemptive intervention or treatment adjustment before functional decline becomes apparent.

Our ablation results (Table 6) demonstrate that voice features consistently improve performance across all 40 cross-validation folds, with the Clinical + Voice + Meta configuration achieving 10-fold lower variability (std = 0.0209) compared to Clinical-Only (std = 0.20). This enhanced stability confirms reproducible contribution despite modest effect size. The paired t-test comparing Clinical + Voice + Meta vs. Clinical-Only yields $p = 0.003$, validating statistical significance.

Future work employing advanced acoustic feature extraction, deep learning-based embeddings from pre-trained audio models, prosodic analysis (pitch variability, speaking rate), and articulatory precision metrics, may further enhance voice biomarker utility. Additionally, integration of high-quality recordings from dedicated apps rather than telephone-based telemonitoring could reduce measurement noise and strengthen voice signal contribution. Our use of “context-aware” refers specifically to the model’s integration of patient-specific longitudinal clinical narratives (text embeddings) that capture individualized disease characteristics beyond scalar measurements. These narratives synthesize multi-visit patterns including symptom variability, progression rates, and vocal impairment severity, providing holistic patient context that informs predictions.

However, an important limitation of this observational study is that our model predicts observed UPDRS trajectories that reflect both natural disease progression and effects of medical interventions (levodopa, dopamine agonists, MAO-B inhibitors, physiotherapy). The Parkinson’s Telemonitoring dataset does not include medication dosing information, treatment initiation/modification dates, or intervention adherence metrics. Consequently, our predictions represent “treated disease outcomes” rather than pure biological progression.

This has two clinical implications:

**1. Prediction interpretation:** A predicted UPDRS increase may reflect inadequate treatment response, disease advancement despite optimal therapy, or medication non-adherence, scenarios with different clinical management strategies. Clinicians must interpret predictions within the context of each patient’s current treatment regimen.

**2. Generalizability constraints:** Model performance may vary for patients with significantly different treatment protocols than those in the training cohort. The model implicitly assumes treatment patterns similar to those in the original telemonitoring study (predominantly levodopa-based management in early-to-moderate stage patients).

Future iterations should incorporate treatment variables as explicit model inputs, enabling:

- Stratified predictions conditional on specific treatment regimens
- “What-if” scenario modeling for treatment adjustment decisions
- Disentanglement of treatment effects from disease progression
- Personalized predictions accounting for medication response heterogeneity

Despite this limitation, our model provides clinically actionable predictions for monitoring disease trajectories under real-world treatment conditions, which is the primary use case for longitudinal PD management tools. Prospective validation studies should collect detailed medication histories to enable treatment-aware prediction modeling.

A critical aspect of our methodological approach is the proper separation of feature types into distinct modalities. we reorganized features into four clearly defined categories: (1) voice biomarkers with signal processing enhancements, (2) clinical progression features capturing disease trajectory, (3) basic demographic/temporal metadata, and (4) semantically enriched text embeddings.

This organization revealed that clinical progression features are the strongest individual predictors, which aligns with clinical intuition, past UPDRS trends naturally inform future scores. However, our ablation studies (Table 6) demonstrate that voice biomarkers and text embeddings provide substantial complementary information.

Critically, our ablation studies show that each modality addition produces performance improvements, with the full four-modality model achieving $R^2 = 0.9925$ compared to $R^2 =9905$ for three-modality combinations. This validates the core premise of our work: that multi-modal integration enhances prediction accuracy beyond what any single data type or subset can achieve.

The Enhanced DCAN demonstrates statistically significant improvements in predictive accuracy, achieving an average RMSE of 0.6727 and R² of 0.9925, outperforming standard algorithms such as Ridge and Random Forest, whose RMSEs were over 1 and R² values under 0.98. The superiority of our method stems from three factors: (1) explicit temporal modeling through bidirectional LSTMs that capture sequential dependencies, (2) multi-head attention mechanisms enabling adaptive weighting of historical observations, and (3) dynamic cross modal fusion allowing synergistic integration of diverse data types. Classical methods, even ensemble approaches like Gradient Boosting, treat flattened sequences as independent samples and cannot leverage temporal structure or cross-modal interactions as e?ectively. This substantial improvement highlights the strength of our novel multi-head dynamic attention fusion mechanism, which enables the model to weigh and integrate heterogeneous modalities flexibly over time, extracting richer clinical insights than static or unimodal methods. Ablation studies and performance comparison further revealed that engineered clinical features and patient summary embeddings carry the bulk of predictive power, while voice and meta features offer complementary, albeit smaller, contributions. Such findings validate prior clinical literature emphasizing longitudinal clinical markers while underscoring the innovative role of natural language processing to capture nuanced patient context. Our architectural choices, stacked bidirectional LSTMs, and dynamic gating, collectively enhance temporal modeling fidelity and interpretability, mitigating common pitfalls like overfitting and temporal leakage in longitudinal clinical AI.

Clinically, these findings have important implications. By providing accurate, continuous predictions of motor symptom trajectories, our model can support early intervention and tailored treatment adjustments, optimizing patient outcomes. The use of non-invasive modalities like voice and routinely collected clinical data enhances feasibility for repeated, remote monitoring, particularly valuable for PD patients with mobility limitations. Additionally, the interpretability afforded by abaltion study and importance change advances clinical trust and may guide neurologists in focusing on patient features most indicative of progression, helping to personalize therapeutic strategies. The modular design and computational efficiency of our approach facilitate integration into telemedicine platforms and electronic health records, potentially enabling scalable, longitudinal PD management and more proactive care models.

Several important limitations warrant discussion and constrain the generalizability of our findings:

**1-** Our evaluation is based on 42 patients with repeated 5-fold cross-validation yielding 8-9 patients per test fold. While patient-level cross-validation with 8 repetitions (40 total folds) substantially mitigates small-sample concerns through repeated evaluation across different patient subsets, the limited cohort size has several implications:

**Confidence interval width:** The standard deviation in cross-validated $\hbox {R}^2$ (±0.0027) indicates good stability, and the 95% confidence interval [0.9916, 0.9934] is exceptionally narrow due to 40 independent evaluations. However, confidence intervals remain moderately wider than large-scale studies with thousands of patients would achieve.

**Subgroup analysis limitations:** Stratified performance analysis for advanced-stage patients could not be robustly computed due to absence of UPDRS >41 patients in the dataset. This prevents assessment of model performance across the full disease severity spectrum.

**Generalization uncertainty:** With only 42 patients from a single telemonitoring study protocol, we cannot definitively assess performance across diverse clinical populations, geographic regions, or healthcare settings. The repeated CV framework provides strong evidence of generalization *within* this cohort’s patient distribution but does not guarantee external validity to fundamentally different populations.

**2-**The Parkinson’s Telemonitoring dataset originates from a single study with specific inclusion criteria (early-stage PD), voice recording procedures (telephone-based), and monitoring protocols (6-month duration). External validation on independent cohorts with different characteristics is essential before clinical deployment:

- **Recording equipment and environments:** Smartphone-based apps vs. telephone; home vs. clinic settings
- **Disease stage distributions:** Including advanced-stage patients with motor fluctuations
- **Demographic diversity:** Varied age ranges, ethnic backgrounds, comorbidity profiles
- **Clinical protocols:** Different UPDRS assessment frequencies, multiple raters with varying experience
- **Treatment regimens:** Complex polypharmacy, deep brain stimulation patients, medication-naive cohorts

**3-** Until such external validation is performed, our model should be considered a proof-of-concept for multi-modal PD progression modeling rather than a clinically deployable tool.

**4-** As discussed in results section, predictions reflect treated disease trajectories without explicit modeling of interventions. This limits causal interpretability and may reduce generalizability to patients with substantially different treatment protocols.

**5-** Our text embeddings rely on synthetically generated narratives from longitudinal statistics rather than authentic clinical notes. While our approach demonstrates that contextualized patient summaries provide value (3.82% RMSE reduction), integration of actual electronic health record narratives, physician assessments, patient-reported outcomes, medication adjustment rationales, could provide richer clinical context. Domain-specific language models (BioBERT, ClinicalBERT) fine-tuned on neurological text may further enhance semantic representation quality.

**6-** Cross-validated ablation studies (240 model trainings: 6 configurations $\times$ 40 folds) require substantial computational resources ($\sim$2–3 hours per configuration on modern GPU hardware, $\sim$12–18 hours total). While this investment is justified for robust modality assessment in clinical applications, it may limit feasibility for rapid model iteration in resource-constrained settings or real-time clinical deployment scenarios.

These limitations highlight the need for multi-center prospective validation studies with larger, more diverse patient populations spanning the full disease spectrum before this modeling framework can be responsibly integrated into clinical decision support systems. The exceptional stability demonstrated through repeated cross-validation ($\hbox {R}^2$ std = 0.0027 across 40 folds) provides strong evidence of reliability *within the studied cohort*, but external validation remains the critical next step for clinical translation.

Looking ahead, future research should prioritize expanding data diversity, especially for advanced PD stages, to bolster model robustness across the full disease spectrum. Methodologically, exploiting self-supervised learning for missing data handling, incorporating additional modalities like genetics or imaging, and refining fusion architectures could further enhance prediction accuracy and interpretability. Efforts to develop patient- and clinician-friendly interpretability tools will be crucial to foster clinical adoption and informed decisiometa-n-making. Prospective, multi-center validation studies and integration into clinical workflows, alongside close collaboration with neurologists and ethicists, will pave the way for responsibly translating these AI advances from research to bedside. Addressing regulatory standards and privacy safeguards will also be essential for ethical, safe implementation.

In conclusion, our dynamic context-aware multi-modal deep learning framework substantially advances longitudinal prediction of Parkinson’s disease progression, leveraging innovative temporal fusion and natural language processing to deliver precise, interpretable, and clinically actionable forecasts. By bridging methodological rigor with clinical relevance, this work contributes significantly to AI-driven precision neurology and lays groundwork for future translational research aimed at improving the lives of those affected by PD.

## Conclusion

In this study, we developed and validated a dynamic context-aware multi-modal deep learning framework for longitudinal prediction of Parkinson’s disease motor symptom progression in early-to-moderate stages. By effectively integrating processed voice biomarkers, engineered temporal clinical features, demographic metadata, and semantically enriched patient summary embeddings through a novel multi-head dynamic attention fusion mechanism, our model captures complex temporal and contextual patterns inherent in heterogeneous PD data.

To ensure robust evaluation despite limited sample size (42 patients), we implemented exceptionally rigorous repeated 5-fold cross-validation at the patient level (8 repetitions, 40 total folds), substantially exceeding standard evaluation practices. Our approach achieves state-of-the-art accuracy (mean $\hbox {R}^2$ = 0.9925 ± 0.0027, RMSE = 0.67 ± 0.19, MAE = 0.50 ± 0.15) with all 40 folds achieving $\hbox {R}^2$ > 0.989, significantly outperforming classical machine learning baselines (most $p < 1 \times 10^{-5}$) and all previously published methods on this dataset.

Cross-validated ablation studies (240 total model trainings across 6 configurations $\times$ 40 folds each) demonstrate that integrating voice biomarkers, clinical progression features, demographic metadata, and semantically enriched text embeddings yields statistically significant performance improvements over unimodal and subset-modal approaches. Clinical progression features establish a strong baseline ($\hbox {R}^2$ = 0.9887 ± 0.0043), while text embeddings provide the largest incremental gain (3.82% RMSE reduction with enhanced stability). Voice biomarkers contribute modestly to accuracy (2.72%) but substantially improve robustness (10-fold lower variability). The full multi-modal model achieves optimal performance (7.50% RMSE reduction vs. clinical-only) with the lowest variability (CV = 0.27%), demonstrating that dynamic cross-modal fusion enhances both accuracy and prediction stability.

The exceptional consistency across 40 independent evaluations ($\hbox {R}^2$ range: 0.9899–0.9947, coefficient of variation: 0.27%), with each patient tested 8 times in different contexts, provides robust statistical evidence of reliable generalization within the early-to-moderate PD spectrum represented in our cohort. This stability, combined with superior average performance, demonstrates that our multi-modal architecture successfully captures generalizable disease progression patterns suitable for prospective clinical validation.

Our approach advances the frontier of precision neurology by delivering accurate and individualized predictions of motor symptom trajectories in early-to-moderate PD, which are critical for timely intervention and personalized treatment planning. The scalability and modularity of our framework make it well-suited for integration into remote monitoring and telemedicine infrastructures, addressing the growing need for accessible, continuous PD management.

While limitations exist, particularly regarding external validity, advanced-stage performance (due to data unavailability), and treatment confounding, this work provides a strong methodological foundation for future studies. Prospective multi-center validation with larger, more diverse cohorts spanning the full disease spectrum, explicit treatment modeling, and integration of authentic clinical narratives represent critical next steps for clinical translation.

By combining rigorous statistical validation, transparent reporting of limitations, and publicly available code, our framework demonstrates the potential of multi-modal deep learning to contribute meaningfully to improved patient monitoring and clinical decision support in Parkinson’s disease management, pending further validation in diverse clinical settings.

## Data Availability

The Parkinson’s Telemonitoring dataset used and analyzed during the current study is publicly available in the UCI Machine Learning Repository at https://archive.ics.uci.edu/ml/datasets/parkinsons+telemonitoring.

## Code availability

In line with the journal’s editorial policies, the custom code used for the Dynamic Context-Aware Multi-Modal Deep Learning framework and its evaluation are publicly available and permanently archived for citation. The complete code, which includes the implementation of the repeated 5-fold cross-validation methodology, can be accessed via the following Digital Object Identifier (DOI): https://doi.org/10.5281/zenodo.17815307.
